# ‘Rule your condition, don't let it rule you’: young adults’ sense of mastery in their accounts of growing up with a chronic illness

**DOI:** 10.1111/1467-9566.12298

**Published:** 2015-07-03

**Authors:** Janet Heaton, Ulla Räisänen, Maria Salinas

**Affiliations:** ^1^ University of Exeter Medical SchoolUniversity of ExeterUK; ^2^ Nuffield Department of Primary Care Health SciencesUniversity of OxfordUK

**Keywords:** adherence, chronic illness, long‐term illness, secondary analysis (qualitative), youth, social theory, accounts

## Abstract

Poor control of chronic illness is often attributed to patients’ non‐adherence to medical advice and treatment. Policy and practice has traditionally focused on improving adherence, assuming that the more patients comply, the better their control and outcomes will be. Drawing on complexity theory, we question this logic in a secondary analysis of qualitative data from studies of young adults’ experiences of growing up with a chronic illness. Examining their sense of mastery of their condition, we found they valued both being in medical control of their condition and having autonomy but had different ideas about how to achieve these goals. While some young adults mostly shared the traditional medical view that achieving good control was the key to retaining their autonomy, others saw control and autonomy as independent, non‐linear and potentially conflicting goals. The latter endeavoured to achieve both goals by striking a balance, variously adopting strategies of engagement with and resistance to their regime in the changing social contexts of their lives. We suggest that policy and practice needs to do more to promote autonomy and adaptive capacity, rather than simply maximising adherence and control, recognising the mundane complexity of living with and managing a chronic illness.

## Background

In his classic study of the lives of people with diabetes, David Kelleher (1988a,1988b) argued that the notion of being in control of the condition can be double‐edged. While following medical advice and adhering to treatment can enable people to successfully control their blood glucose levels and stay well, it can also engender a feeling of a loss of freedom and control over their lives, and be associated with a ‘reduced sense of autonomy in everyday activities’ (Kelleher 1988a: 27). Kelleher suggested that this might explain why, as he observed, some people do not always fully adhere to their regime, attaching a higher priority to maintaining a ‘normal’ identity instead. Subsequent qualitative research has shown that, despite historical changes in how diabetes is treated, people continue to adopt apparently paradoxical strategies of engagement with and resistance to their therapeutic regime (Campbell *et al*. 2003, Ingadottir and Halldorsdottir 2008, Paterson *et al*. 1998).

Whether people with diabetes and other chronic conditions do or do not adhere has been increasingly understood in relation to their concern with balancing different priorities, rather than their failure to follow medical advice and conform to their therapeutic regime (Campbell *et al*. 2003, Conrad 1987, Donovan and Blake 1992, Paterson *et al*. 1998). This concept of balance was, for example, found to be the ‘predominant metaphor’ of the lived experience of diabetes in a meta‐ethnography of 43 qualitative research reports (Paterson *et al*. 1998: 58). It was echoed in a later synthesis of seven articles on the same topic, in which the researchers generated a model of patients’ approaches to managing their diabetes based on their notions of balancing their health and well‐being (Campbell *et al*. 2003). More recently, researchers have drawn attention to the ‘dynamic, iterative and balancing process’ of how patients cope with arthritis (Grønning *et al*. 2011: 1425).

While the above research has concentrated mainly on adults and older people, a growing number of studies on younger people who have a chronic illness have reported similar findings. For example, research on children with asthma has shown that they and their families were actively engaged in maintaining a sense of their own ordinariness (Prout *et al*. 1999: 138), and that teenagers with the same condition used strategies to not let the disease get ‘the upper hand’ in their lives (Rydström *et al*. 2005: 388). A study of young people aged 10 to 19 who had thalassaemia major highlighted the ‘constant tension’ that the participants felt about their chelation therapy (Atkin and Ahmad 2000: 510). They saw using an infusion pump to be vital to their health but also restrictive and damaging to their identity, and they tried to minimise this damage. The researchers claimed that achieving this balance was part of a ‘dynamic process’ that explained why the young people's responses to chelation therapy were ‘constantly shifting and at times appeared contradictory’ (Atkin and Ahmad 2000: 509).

Despite these findings, few studies have purposefully set out to explore what it means to people to control and master their chronic illness, and how this relates to the ways in which they self‐manage their condition. In a notable exception, Ingadottir and Halldorsdottir (2008: 608) explored the ‘essential structure of mastering diabetes from the patient's viewpoint’ in a small qualitative study of adults’ experiences of adhering or not adhering to the therapeutic regime for their insulin‐dependent diabetes. They found that patients’ ideas about control differed from those of health professionals. Patients talked of their daily struggle to achieve a balance between the freedom and constraints of the regime. They felt that their personal autonomy was challenged both by strict adherence to the regime and by the risk of complications from not looking after their condition well enough, posing a dilemma that they had to constantly negotiate. Most of the participants modified or adhered selectively to their regime in trying to strike a balance.

In addition, previous work elaborating people's various styles of adjustment to chronic illness (Radley and Green 1985), and the strategies by which they variously adopt, adapt or resist therapeutic regimes, has mostly been carried out in studies of single conditions. There has been relatively little cross‐cutting research examining how people's responses to illness vary across condition groups and fluctuate over time. The shifting perspectives model of chronic illness, developed by Barbara Paterson (2001), provides some redress. It was derived from a meta‐study of over 250 qualitative research reports on the experiences of adults with chronic physical illness. In the model, the ways in which people experience illness is represented as a complex dialectic between individuals and their always changing worlds. People move between states of ‘illness in the foreground’ and ‘wellness in the foreground’ where their views on their situation alter over time (Paterson 2001: 23). The model provides a useful conceptualisation of the shifting nature of people's responses to chronic illness and the non‐linear nature of illness trajectories. However, it provides a limited theoretical understanding of the underlying mechanisms by which people variously and continuously adapt to their illness, and how they can be better supported by services to this end.

In this article, we sought to build upon the above literature in two ways. Firstly, relatively little is known about how young adults who have grown up with a chronic illness conceptualise achieving a balance between the freedom and constraints of their medical regime. The present study was specifically designed to explore this. Drawing on a large and diverse sample of young adults with various long‐term conditions, we examined what we refer to as their ‘sense of mastery’ of their condition, or what it meant to them to control their condition without it controlling them. Secondly, we attempt to provide a fresh perspective on the topic by drawing in the analysis on concepts and tools from complexity theory, which we outline below. We hope that the application of this perspective helps to shed some new light on the variable adaptive responses of people to chronic illness and, in particular, why people do or do not adhere to their medical regime.

### The relevance of complexity theory

Complexity theory was developed as an alternative paradigm to traditional Newtonian scientific thought, where the world and its sub‐systems are viewed as if they are machines that operate like clockwork (Kernick 2004, Zimmerman *et al*. 2008). Rejecting this metaphor, complexity theorists differentiate between simple, complex and chaotic forms of behaviour, each of which is founded on different rules of interaction. The multidisciplinary science of complexity is itself principally concerned with the study of complex forms of behaviour characteristic of complex adaptive systems (CAS) in the natural and social world. CAS operate in emergent rather than mechanical or irregular ways (Zimmerman *et al*. 2008). Weather systems, eco‐systems, communities, healthcare organisations and doctor–patient interaction are all examples of CAS. These systems are nested in and operate alongside other CAS in the local and wider environment they share. For example, in England, hospital trusts are CAS nested within the National Health Service (NHS). They operate alongside other CAS in the form of primary care trusts and other health and social care organisations. These systems also consist of other CAS in the form of patients, clinicians, managers and support staff, whose micro‐interactions help to shape the macro‐systems they are part of.

One of the defining features of CAS is that they have the capacity to self‐organise, or internally evolve in response to changes in other CAS in their environment. Because self‐organisation is a distributed and not a centralised process, each system has a degree of freedom to develop and adapt in relation to other systems. The ways in which each system adapts in its environment in turn influences and shapes the ways in which other CAS co‐evolve over time. A key point is that CAS evolve in a state of constant tension and balance. They thrive in a state of ‘bounded instability’ on the edge of chaos where they have time, connections and means to adapt to the nature and pace of change in their environments (Zimmerman *et al*. 2008: 12). Conversely, they stagnate in conditions of order or stasis and dissipate in conditions of disorder and turmoil. Through the tensions that build from interactions with adjacent systems, CAS co‐evolve over time through self‐organisation, dynamically shaping and being shaped by their environment. Change is an emergent property of CAS. Tension, dynamism and innovation are the hallmarks of a healthy system, rather than equability, stability and inertia.

CAS possess a number of other characteristics, which have been comprehensively described elsewhere (Kernick 2004, Zimmerman *et al*. 2008). These include the property of being shaped by interactions that are non‐linear, whereby small actions can have big effects and vice versa. CAS are also history‐dependent or path‐dependent, which is to say they are shaped by where they have been. Agents in social systems also have the capacity to learn from their experience, adapting the strategies by which they interact with other systems in their ever‐changing environment. Because of their emergent qualities, the outcomes of CAS are difficult to predict with certainty and to direct through interventions. However, the behaviour of CAS are governed by simple rules. These rules guide (but do not determine) the behaviour of CAS within observable parameters of time and space, creating so‐called ‘attractors’ that reflect the overall dynamical state of each system.

In applied health research, complexity theory has been used for a range of purposes, from the study of innovation in healthcare organisations (Kernick 2004, Plsek *et al*. 2002) to consultations in primary health care (Innes *et al*. 2005). However, it has seldom been used to examine patients’ experiences of illness. Helen Cooper and Robert Geyer (2007, 2009) have shown the potential for this application in their work on the medical management of diabetes. Cooper (who has diabetes herself) and Geyer argue that traditionally, the aim of diabetes management in the English NHS has been to enable patients to achieve and maintain tight control of blood glucose levels and that poor control has been generally attributed to their poor management or non‐adherence. Challenging this linear perspective, they argue that diabetes is a complex problem, involving the interaction of many unpredictable factors, rather than a simple problem that can be managed by maximising adherence.

**Figure 1:**
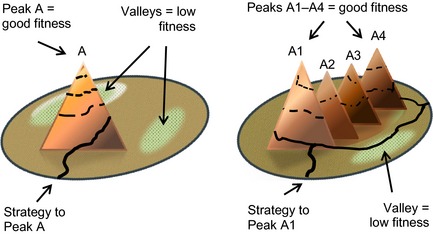
Contrasting fitness landscapes.

Among the tools Cooper and Geyer use to illustrate the complexity of managing diabetes are fitness landscapes. This tool was originally developed by the population biologist Stuart Kauffman (1995) to model the way that species variously adapt to their changing environment over time. In Figure 1 the single‐peak landscape on the left represents the traditional medical approach to managing diabetes, which is focused on a single goal (A) – achieving tight control – and the primary strategy for achieving this ‘fitness’ peak is via adherence. This contrasts with the more rugged landscape on the right which, Cooper and Geyer argue, represents the reality of people's experiences. Here people have to make choices and adopt strategies that take them higher towards peaks of good control (A1–A4), or lower towards valleys of poor control, before ascending and descending again. The courses that people take may vary, some steady and some uneven, depending on the fit of their strategies with their environment. Cooper and Geyer argue that the goal of diabetes management should be to enable people to develop the capacity to continuously adapt to this rugged landscape, where swings rather than stability are the norm, and which itself changes over time, rather than purely striving for constant perfect control.

It should be noted that while blood glucose control is the main goal in both these landscapes, there is in principle no limit to the number of goals that could be included, introducing yet more complexity into the model as people have to choose between and develop strategies for meeting multiple priorities. In our analysis we draw on and extend some of the ideas from complexity theory to examine young adults’ sense of mastery of various long‐term conditions while traversing the equivalent of a contemporary fitness landscape for children, young people and young adults.

## Aims and methods

### Design

Secondary analysis has been increasingly used in qualitative research to carry out studies based on existing data (Heaton 2004). In this secondary study we draw on interview data from a collection of national studies carried out by the Oxford Health Experiences Research Group (HERG), which were designed to capture and share people's real‐life experiences of medical conditions and health care in the UK. An integral part of the studies is the dissemination of the findings via the www.healthtalk.org website, so that other patients and their families, as well as the public and healthcare professionals, can learn from the participants’ first‐hand accounts. The website contains thematic summaries of key topics, supported by video, audio and/or written extracts from the interviews, with participants’ consent. The full interview transcripts are anonymised and archived by the University of Oxford, where they are available by request for use in secondary studies, subject to a licence agreement.

After the first author scoped the data in the archive, she discussed her potential ideas for secondary research topics with the primary researchers who had conducted the studies of interest (Heaton 2014a). This resulted in the identification of the topic for the present secondary study, which was designed to investigate the following questions: what did it mean to young adults to control and master their condition? Did they claim to have ever achieved this sense of mastery? How did they account for achieving a positive or negative sense of mastery over time? The secondary study was designed and led by the first author, and the analysis for this article was carried out in collaboration with the co‐authors who carried out the interviews in the primary studies.

### Sample

The HERG studies of younger people's experiences of diabetes (type 1), epilepsy and a range of long‐term conditions were purposely selected to facilitate an analysis of young adults’ sense of mastery within and across multiple condition groups. These three studies were carried out in 2006–2008. Each study sample was diverse in terms of age, gender and length of experience of the condition; the epilepsy study also included a range of subtypes of the condition. Two participants were excluded from the secondary analysis because they had developed their condition after the age of 19 and so had no experience of growing up with a chronic illness through childhood or adolescence. This left a final sample of 102 interviews with 103 people (one was a joint interview with twin brothers). Of these, 42 were male and 61 female; 40 had epilepsy, 39 had diabetes and 24 had other long‐term conditions (including juvenile arthritis, cystic fibrosis, asthma, eczema, sickle cell disease, scoliosis and others). The participants were aged 15–29 years at interview (an average of 20 years of age) and 0–19 at onset of their condition (an average of 10 years of age); they had lived with their condition for 1–25 years (an average of 11 years from reported onset).

### Interviews

In the interviews the participants were initially invited to speak freely about their experiences of living with their condition. They often began by explaining how they had learned that they had their condition before moving on to discuss what it was like living with it through childhood and/or adolescence. The interviewer then asked questions using a semi‐structured topic guide. This included similar topics across the three studies, reflecting the common purpose of the studies. The participants were, for example, usually asked if they had any advice or messages for others who had the same condition, and for professionals who care for them.

### Analysis

A combination of thematic (across case) and narrative (within case) analysis was used along the lines described by Flick (2006). Case‐by‐case summaries of the biographies were prepared, with key themes relevant to the research aims noted in the process. Initially, a coding framework with 13 broad codes was developed and used to index the transcripts using Atlas.ti. Additional sub‐coding of selected material was carried out successively by hand where required. A thematic analysis was conducted using a series of charts summarising participants’ views and experiences across the sample on particular themes. The coding and charting was carried out by the first author and checked by the co‐authors. A selection of cases derived from the thematic analysis was then subjected to narrative analysis, exploring the ways in which individuals accounted for the development of their sense of mastery over the course of their illness trajectory. Here, because of space constraints, we concentrate on the results of the thematic analysis. Early findings were discussed at a workshop with a group of young adults with chronic illness, and with members of our advisory group for the study, and their comments helped us to refine the analysis for this article.

We turned to complexity theory in the course of the analysis, while we were examining the young adults’ ideas about how different states of (positive and negative) mastery had been or could be achieved. It was then that we were struck by the ways in which many of their accounts of how they had adapted to their illness were consonant with a complexity view of the world. We proceeded to develop our analysis of the young adults’ sense of mastery of their condition using some of the concepts and tools from complexity science.

### Ethics

The primary researchers had obtained informed consent from the participants in writing prior to the interviews. This included their permission for the anonymised transcripts to be archived for use in further research or teaching. The participants were also given the opportunity to check and edit their transcripts and approve the final versions. Formal ethical approval for the above was obtained by HERG from a NHS multi‐centre research ethics committee. Additional ethical approval for the secondary study was sought from a university research ethics committee but after the protocol was reviewed we were informed that it was not required.

## Findings

As the preliminary scoping analysis had suggested, the young adults in the three studies often talked about their experiences of growing up with a chronic illness in terms of whether they felt they controlled their condition or it controlled them. In their advice to others, some of them also said it was to ‘rule your condition, don't let it rule you’ or sent a similar message to their peers. Below we describe our analysis of what it meant to the young adults to rule their illness and how they accounted for achieving this sense of mastery or not.

### A sense of mastery

The young adults expressed their mastery of their condition in two ways: firstly, in terms of whether they thought they had achieved good medical control of their condition and, secondly, whether they felt that their autonomy had been compromised by their illness. The overall sense of mastery that they conveyed in their accounts depended on whether they expressed a largely positive or negative view of their present control and/or autonomy. They defined their levels of control and autonomy as follows.

#### Control

The young adults commonly talked about how well or not well their condition was controlled medically at the time of the interview, and how this compared with their previous levels of control, showing stability, improvement or decline over time. Clinical markers and reference points were cited as benchmarks of their stability and level of control. For those with diabetes, typical indicators were their range of daily blood glucose scores and their average scores in the form of their periodic HbA1c tests. For those with epilepsy, it was the frequency, type and severity of their seizures. And for those with other conditions, it was how often they had flare ups (arthritis), chest infections (cystic fibrosis), attacks/crises (sickle cell disease) or pain (various conditions) and had been hospitalised, or had time off school, university or work. In describing their current status in these terms, the young adults often commented on whether they were content with their level of control and how easy or hard it was to maintain. Whether they presented a mainly positive or negative view of their control depended on how acceptable the level of control that they had attained was to them.

Related to this, whether the young adults were able to achieve this medical control for themselves, or if they had to rely on others for help, was also important to them. Many had reached a point where they felt they were responsible for controlling their condition themselves, rather than their parents or doctors. Some had taken a lot of responsibility from a young age, whereas others had relied on their parents for longer – particularly if they had experienced issues such as feeling unable to inject themselves or finding it hard to remember when to take medications. Taking on responsibilities for controlling their condition themselves was generally represented as a positive indicator of their growing independence and command of their condition. In some cases, the participants who had done this reported that their level of medical control had improved since they took over, although in others they observed that it had declined.

#### Autonomy

The young adults also talked at length about whether or not the advent of their condition had stopped them from living what they called a ‘normal’ life for their age or had changed them into someone defined by their illness. They saw their condition as a potential threat to their autonomy or freedom to live their lives unconstrained by their condition. In their accounts they often used social comparisons to illustrate how their lives had or had not been changed by their condition (Heaton 2014b). For example, some compared their lives to their non‐disabled friends and peers to show that they had managed to do the same things as them. Some also compared themselves to their pre‐onset of condition selves to demonstrate that they had either managed to pursue their personal interests unabated or that their previous lifestyle and plans had been disrupted.

Many claimed that, despite their illness, they had been able to do so‐called normal things such as play sports, go to university and maintain a social life, as well as to do some exceptional things such as climbing a mountain. However, many also reported that there were activities that they had not been allowed to do, such as participate in some school activities, go to sleep‐overs away from home, drive a car or motorbike and spend time alone. Some had also been denied the opportunity to apply for certain jobs or had lost jobs or been unable to find work due to their condition. Whether the young adults presented a largely positive or negative view of their autonomy in their accounts partly depended on the range and personal significance of the particular things that they had or had not managed to pursue; for example, wanting to drive or join the army was more important to some than it was to others. It also depended on whom they elected to compare themselves with and whether they made downward, upward or lateral social comparisons to these particular reference groups.

#### Varying and shifting sense of mastery

Across the sample there was considerable variation in the young adults’ views of their present levels of control and autonomy, and in the overall sense of mastery that they conveyed in these terms. Some of the participants portrayed a broadly positive view of both their control and their autonomy. They were content with their medical control and did not feel that their condition had stopped them doing what they wanted to do in their young lives to date. In contrast, others had a mainly negative view of both. They felt out of control of their condition and claimed that it had prevented them from doing some things that were important to them. Yet others had a positive view of their control but a negative view of their autonomy, or vice versa. In the former state, despite being in medical control of their condition they felt that their lives were still shaped by and at times even overtaken by it. In the latter, they had rebelled against their regime and neglected their control, continuing to live as if they did not have the condition as far as possible.

In many cases, the young adults also described a shifting sense of mastery. They explained how they had gained or lost medical control of their condition over time, or how they had gone through a rebel phase where they prioritised living a normal life over managing their condition, or how they at times felt that they were ‘losing themselves’ to their condition. However, there were also some participants, such as those with less severe forms of diabetes and asthma, who described how they had quickly and progressively gained control and stayed stable, reporting no side‐effects and little disruption to their lives to date. Likewise, there were also some participants who seemed trapped in a negative state of mastery, lacking control of their condition and autonomy in their lives. They included some with intractable epilepsy who had not found a treatment that worked, and some with diabetes who had regularly abused their insulin while also having an eating disorder. Later we look in more detail at the changes in the young adults’ lives that affected their levels of control and autonomy over time and how they responded, as well as how they made changes to try and achieve these goals. But first we examine their ideas and underlying theories about how a sense of mastery could be achieved.

### Perspectives on achieving mastery

While having medical control and retaining autonomy were both important to the young adults, we found that they had different ideas about how these goals were related to each other and how they had been or could be achieved. As we show below, the young adults variously drew on and promoted orderly (linear) and complex (non‐linear) theories of the relationship between control and autonomy in their accounts and the associated strategies for achieving these goals.

#### Orderly perspective

At times, some of the young adults expressed a relatively simple, orderly and linear view of the relationship between the goals of control and autonomy, and how these could be achieved. They believed that by gaining and maintaining good medical control of their condition they would be more likely to stay well and so resist the threat it posed to their autonomy. This outlook is illustrated by the following excerpt, where a young woman explains her motivation for looking after her diabetes:

Just to remain in good health. I don't want any problems like blindness or things like that. I don't want any problems with my diabetes. So to have good control and look after myself is vital if I'm going to remain in good health. And also because I've got a lot of plans for my future, [um] what I want to become, all things like that. It's important. Like I'm, I'm only just beginning my life. I need to keep my diabetes controlled so I can fulfill all what I want to do with my life.

(D33/409, age 18)

Likewise, they thought that if they lost control then they would lose their autonomy too. This view is illustrated by the following excerpt, where a young man with asthma advises others with the same illness to:

Just keep it under control really. It's better to have it under control and being able to do anything you want in your life than not bothering and then regretting that because you couldn't do something you've really wanted to do.

(C09/548, age 16)

Thus, from this perspective, having or retaining autonomy was largely seen to be a corollary of staying in good control of their condition.

The achievement of good control, and ergo autonomy, was in turn seen to be related to how well the young adults had adhered to their regime and followed medical advice. Good control was variously attributed to the adoption of good management practices, such as prioritising their illness, following the regime and medical advice, having an effective intervention that worked well as long as they followed it properly and having appropriate support from services, family and friends. Poor control was seen to result from a deficit in the above, as a result of not paying sufficient attention to their condition, finding their regime hard and difficult to follow, medications not working or having unacceptable side‐effects and not having help from others to manage their condition effectively.

**Figure 2:**
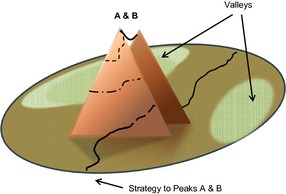
A fitness landscape with control and autonomy as aligned goals.

Underlying this orderly perspective was the idea that the more closely they adhered to their therapeutic regime and followed advice the greater their control and their autonomy were likely to be. The relationship between the strategy of adherence and the goals of control and autonomy was seen to be linear, where the work people put into managing their condition was expected to lead to a proportional increase in both control and autonomy. Conversely, failure to adhere to medical treatment and advice was associated with a corresponding loss of control and decline in autonomy. This view of the relationship between control and autonomy is shown as a fitness landscape in Figure 2. Here the mountains of control (A) and autonomy (B) are coterminous, with the goals aligned and converging to form a massif, where both goals can be reached via a single pathway or strategy (adherence) leading to a close horseshoe arrangement of peaks.

While a minority of the young adults’ accounts provided unequivocal support for the orderly view of the relationship between control and autonomy, others promoted a qualified version of it, based on their experiences of following the approach. For example, a few participants with diabetes explicitly stated that they had sought to achieve ‘perfect control’ of their condition through close adherence to their insulin regime, but none claimed to have actually achieved this goal for very long. They described how they had gone through a ‘honeymoon period’ early in their treatment. This was when their insulin had taken effect and they felt a lot better, and they had no difficulties in keeping good and stable control. But after this period they had started experiencing swings in their blood glucose levels and, much to their frustration, found that they could no longer achieve medically ideal scores consistently. They had come to realise that aiming for perfect scores all the time was stressful and unrealistic and they had learned to be more relaxed about what was attainable and acceptable. As one participant opined, the ideal blood glucose score was ‘between 4 and 10’ before cautioning: ‘you were never going to be a perfect diabetic person’ (D18/149). In these ways, their initial linear belief that close adherence would enable them to achieve perfect control had been challenged by their actual experience and they had adjusted their expectations accordingly.

#### Complexity perspective

In contrast to the above orderly perspective, it was more common for the young adults to express a complex and non‐linear view of the relationship between control and autonomy and the strategies by which these goals could be achieved. From a complexity perspective, the attainment of good control was not necessarily seen as imperative and synonymous with autonomy. Instead, the young adults were more ambivalent about these goals and how to achieve them. This is illustrated by the following excerpt, where a young woman states that it was important to her to manage her diabetes properly but:

Not to be sort of obsessive and meticulous about it, not to do it so much that it kind of controls your life and that you're always thinking about it … but in as much as possible while still being kind of normal to just kind of you know try and keep it within certain boundaries of control.

(D7/727, female, age 20)

Similarly, another young woman with epilepsy advises others to be proactive and deal sensibly with their condition but at the same time not to let these goals take over their life and totally define them:

… [T]ry and find out as much information as they can … just not to let it, not to let it take over too much as well, not to let it take over who you are, because I think if you do that then it's gonna take over, it can take over your life and that's, ‘cos epilepsy is part of you, you are not part of epilepsy if that makes sense … . So I would say … you've still got to be sensible and do, and deal with your epilepsy in a sensible way, but still go ahead and try and do the things that you wanted to do anyway.

(E9/522, female, age 23)

As these excerpts show, there was a real concern that too close a focus on gaining control might be counter‐productive for their autonomy. From this complexity perspective, control and autonomy were seen to be independent and potentially conflicting goals, which they had to separately prioritise.

Rather than seeking to achieve control and autonomy through the single strategy of adherence, the young adults advocated balancing the work they put into controlling their condition on the one hand, with the work they put into resisting its impact on their autonomy on the other:

It [diabetes] does change your life but it's not in a, it doesn't have to be in a massive way if you can just get the balance right of just spending enough of your time managing it but not letting it rule your life.

(D13/599, male, age 19)

I mean you can't be an angel all the time, sometimes you, if you look after, and you do eat healthily, you do all your exercises all the time, you'll get too boring. So it's about striking a balance.

(C1/540, female, age 22, scoliosis)

The ways in which most of the participants described living with their condition was consistent with this outlook, as they variously shifted their priorities and adopted strategies of engagement with or resistance to their regime in pursuit of their respective goals of control and autonomy.

This was evident in the young adults’ accounts of how they selectively adhered to their regime. Examples included: taking their insulin but not doing their blood tests as often as they should; missing insulin injections at lunchtimes in school in order to stay with their friends during these breaks; skipping medication for epilepsy and other conditions; and by taking ‘short cuts’ with their therapy when they could. They also continued to do some activities that they enjoyed – such as drinking alcohol, eating sugary food and staying up late – even though they acknowledged that these were not always in line with medical advice. Taking some risks, occasionally ‘overdoing’ things, and not being a ‘saint’ or an ‘angel’ all the time, was justified if it meant that they could sometimes ‘have fun’ and be ‘normal’. This view was exemplified by the ways in which participants with chronic fatigue syndrome actively prioritised control most of the time but who, by ‘pacing’ themselves, allowed themselves to occasionally prioritise their autonomy and have some fun, even if it was at the expense of their control and they knew they would ‘pay for it’ afterwards.

**Figure 3:**
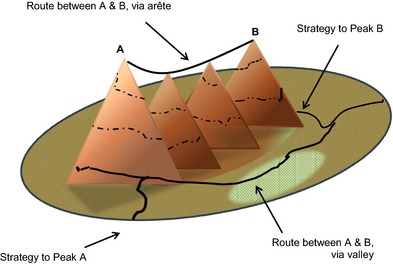
A fitness landscape with control and autonomy as non‐aligned goals.

This complex and non‐linear view of the relationship between control and autonomy and the need to balance strategies for achieving them is shown as a fitness landscape in Figure 3. Here, control (A) and autonomy (B) are represented as separate mountains. The peaks of these mountains are further apart, and in this case separated by an arête and surrounded by deep valleys. Different strategies exist for achieving control (via adherence) or autonomy (via resistance). Some routes take people closer to one goal but simultaneously move them further from the other, while other routes stay relatively close to both or are more circuitous. Each presents different challenges for those traversing the landscape and those involved in their care.

While some young adults had managed to find a balance in the work they put into controlling their condition and their endeavours to live a normal life, others had found it hard, like this young woman with cystic fibrosis:

I just sometimes find it really hard to get a balance between my social life, between my medication and like managing CF. Between my diet and making sure that I eat a good high calorie diet to keep my energy levels up and between my studying. And I just find all of it sometimes hard to balance. And just as I think I've got it all balanced and I think everything's going really well something will happen and then I'll be like, ‘Oh gosh’ like and I have to reassess everything. And I kind of learnt through what I can and can't do and I've learnt through you know, the experience of it all. But I still feel like, even though I've experienced, even though I feel like I know what I can and can't do I still feel like something, sometimes things happen and I think that I haven't really got the right balance but it can be quite difficult. But overall like I do enjoy Uni so it's, it's worth it. [laugh] I hope.

(C15/271, age 19)

Other participants also described times when they felt they had a better balance than others. In the next section we examine some of the changes that the young adults experienced in their lives while they were growing up and how they adapted in response to try and attain a better fit with their goals of control and autonomy.

### Mastery in a changing landscape

As we show below, many of the young adults described how their bodies and lives had changed while they were growing up and how they had positively or negatively adapted in response. Some had also experienced periods of stagnation when their lives were not changing on a par with their peers, which they had endeavoured to overcome by making changes to their environment.

#### Changes in bodies and lives

Many of the young adults reported changes in how their bodies had reacted to their medications over time as they had physically grown and developed. Some of those who had grown up with their condition through childhood and adolescence observed how their condition was fairly stable until puberty, when they then found that their treatment no longer worked as well as it did. Some of the young women had found it harder to control their condition during menstruation, and that some hormonal contraceptives interacted with their other medications. Those who wanted children or who had become pregnant were concerned about the possible effects of their medications on their baby. A number of the young adults had also experienced unwanted changes in their bodies as a result of side‐effects of their medications, including weight gain, nausea, drowsiness and personality changes.

The young adults also experienced a number of life changes as they transitioned to new environments where they had to learn to self‐manage their condition in a different social context. These transitions included changing school, going to college or university, moving out of the family home and living independently, shifting from paediatric to adult health services and entering paid employment. With these changes came new opportunities and experiences: to drink alcohol, cater for oneself, have sex, try illicit drugs, learn to drive and go travelling abroad. For some, these changes had also entailed losing old and gaining new networks of support from family, friends, teachers and healthcare professionals. In addition to these transitions, some of the young adults also described experiencing significant transformative events or ‘wake‐up calls’, such as a medical crisis or becoming pregnant, which they claimed had prompted them to re‐evaluate their priorities and change their behaviour in some way.

Although many of the young adults’ accounts were marked by such changes, it is also important to note that, for some participants, it was a lack of change that was a feature. For example, some of those who had epilepsy from a young age had hoped, as their doctors had told them, that they might grow out of it in time, but this had not happened by the time of their interview. Some had tried various medications but their condition had remained intractable. And some described how, compared with their peers, they had not been able to make some transitions, such as leaving home or learning to drive, because of their condition.

#### Responses

The ways in which the young adults endeavoured to promote their control and/or autonomy in these circumstances was characteristic of the self‐organising behaviour of CAS. As we show below, they interacted with other CAS in their environment, including family, friends and healthcare professionals, adapting to the changes in their bodies and their lives, making changes to their environment and learning through their own and others’ experiences of living with and managing a chronic illness. Through sharing their stories they, in turn, perpetuated this knowledge and so potentially helped to shape the environment for others. Their individual self‐organising responses varied depending on the changes they experienced, the support and options they had to draw on and mobilise in their local environments, and their personal priorities, although control and autonomy were commonly shared goals.

One way in which many of the young adults had adapted to change, or tried to instigate a change, was by moving to a new therapeutic regime where alternatives were available. Some moved to a new drug regime to try and achieve better medical control of their condition or to achieve control with fewer side‐effects, or to achieve a better fit with their lifestyle by reducing the burden of treatment. For example, a number of participants with epilepsy had tried various doses and combinations of medications and a few had undergone surgery. Several with diabetes had switched from a regime of two injections per day at fixed times, with set mealtimes, to a more flexible regime of injecting around the times and type of food they ate, using a combination of fast‐acting and slow‐acting insulin. A few had moved onto a pump that was attached to their body for 24 hours a day and that continuously infused insulin as programmed. In some cases, after making a change, the participants had reverted back to their old regime because they found it more acceptable or they had stopped taking their medications altogether because they found living with seizures preferable to living with the side‐effects of the medication.

As they changed where and with whom they lived, studied or worked, another way in which the young adults adapted to their new situation was by deciding whether or not to disclose their condition to their new friends and colleagues when it was not obvious. In some cases they decided not to tell others because they wanted to be treated the same as their peers or because they were worried it might jeopardise their employment. In other cases they chose to tell key people so that they knew and could provide support if it was needed. For example, some participants had family, friends or key associates who recognised when, unbeknown to them, they were about to have a seizure or were showing signs of hypoglycaemia or hyperglycaemia and could warn them. Some also found that having support from family and friends helped them to feel normal and to accept their illness. Likewise, some had also found that meeting others with the same condition through support groups had helped them to feel less isolated. However, for others the idea of being part of such a group was anathema because it made them feel different and defined by their condition.

Some of the young adults, particularly those with uncontrolled epilepsy, sought to overcome inertia in their lives by changing their environment. For example, a young woman opted to have surgery in a bid to try and stop seizures from her life‐long epilepsy. A young man who, along with his family, recognised he was stuck in a rut with no control over his epilepsy and little autonomy in his life, had moved out of the family home to live independently in a house that had been made safe for when he had a seizure. Another young woman with the same condition was looking forward to finding somewhere to live independently of her parents, who she felt were overprotective, so that she could live more freely.

The young adults’ accounts also demonstrated the ways in which they had learned to manage their condition to promote their control and/or autonomy and the importance they attached to this capacity. A number of the participants emphasised that everyone's experience of illness was different and that everyone had to learn for themselves how their condition affected them and how best to manage it. They claimed that they had learned from their own lived experience, or ‘trial and improvement’ as one young man put it, how their bodies reacted to stress, sleep, food, activity and medications; what their trigger factors and personal limits were; and what risks they could take without losing control of their condition. They used this knowledge to guide their behaviour and to justify their own approach and risk‐taking. Linked to this, some of the young adults were also dismissive of professionals’ advice on the grounds that it was from ‘textbooks’ and neither realistic for young people nor personalised to their own situation. Most had not discussed with their doctors the interaction of their medications with alcohol, contraceptive medications or illicit drugs, relying instead on their experience to guide them.

As well as learning from their own experience, some participants claimed that their approach to managing their condition had been shaped by their knowledge of others with the same illness. For example, one young woman rejected her grandmother's approach of prioritising her diabetes and living her life around the condition. Another young man reported that hearing a friend's experience of drinking alcohol while on epilepsy medication had prompted him to try this for himself. Others reported seeing people who had developed complications and how this had made them consider whether they were doing enough to control their condition in the long run.

## Discussion

This study has added to existing work on patients’ experiences of chronic illness by examining in depth what it meant to young adults to control and master their chronic illness and how this relates to the ways in which they self‐managed their condition while they were growing up. We have argued that, for them, a positive sense of mastery was associated with achieving levels of control and autonomy that were acceptable to them. A negative sense of mastery was associated with not achieving these goals or achieving one at the expense of the other. While the young adults generally valued control and autonomy, they had different ideas on how these goals could be achieved. Some expressed the orderly belief that autonomy was contingent upon good control, which was achieved through close adherence to medical advice and treatment. This view is in keeping with traditional medical models that promote this approach. However, the predominant view was that control and autonomy were independent, non‐linear and potentially conflicting goals that they had to prioritise and balance separately as their lives changed over time. This outlook is, we suggest, more compatible with a complexity view of the world, which we have attempted to render here using some of the more formal concepts and tools of complexity science.

Our application of complexity theory supports and extends Cooper and Geyer's (2007, 2009) work on medical models of managing diabetes by examining how patients’ accounts of their experiences of diabetes and other long‐term conditions provide insights into the mundane complexity of life growing up with a chronic illness. As we have shown, many had found that attaining acceptable levels of control and autonomy was not easy and was influenced by changes in their bodies and lives that they had to adjust to. They adopted different strategies to these ends. Many tried to achieve a balance through partly engaging with and partly resisting their regime, while some tried to adhere more closely to their regime. The former approach had the advantage of enabling them to constantly adjust their strategies to restore a balance when it was upset by change. It also allowed some to at least try and achieve autonomy in the absence of control. The latter enabled some people to achieve control and autonomy in a relatively stable environment. However, as we saw, some found it difficult to sustain ‘perfect control’ when they encountered change, and others found that achieving control by good self‐management did not necessarily result in greater autonomy.

This work has also highlighted the role that people's narratives of their experiences potentially plays in capturing and conveying the mundane complexity of living with a chronic illness and preparing others for this reality. This was exemplified by the young adults’ emphasis on the individuality of their experience and the importance of learning from their own experiences. It was also apparent in their dismissal of professional advice as being too generic and not personalised enough to their young lives and personal situation. Indeed, their messages to others that ‘everyone's experience of illness is different’ and ‘to rule your condition, don't let it rule you’ might be regarded as examples of simple rules or heuristics that the young adults had learned through their experiences and that they were passing on to others through their accounts.

A limitation of the study is that we examined only young adults’ accounts of their experiences and approaches to managing their illness and not observations of their actual behaviour. However, as others have long argued (Bury 2001, Radley and Billig 1996), the study of narratives is important in showing the connections between the ways in which people account for their individual experiences of illness and health care, and the prevailing discourses and moral attitudes of the time. In this case, we have argued that the young adults’ perspectives reflect wider orderly and complex theories of the ways in which medical conditions can be controlled and autonomy retained. While the former reflect traditional medical approaches to managing chronic illness, the latter is grounded in people's lived experience of chronic illness. We have also suggested that their narratives provide a possible mechanism for preparing others for the uncertainty of the lived experience of chronic illness and sharing strategies for achieving acceptable levels of control and autonomy in this context.

### Implications

In this study we have tried to go beyond existing descriptive taxonomies of people's differential responses to illness by using concepts and tools from complexity science. By examining how people act as CAS, complexity theory provides a potentially useful way of understanding how people's responses to illness vary and shift over time, and how they are shaped by and shape their environment. As we have shown, it has provided insights into how and why young adults use different strategies to try and achieve their goals of control and autonomy. More generally, it potentially provides a way of integrating existing and developing new cross‐cutting research on how people adapt to chronic illness in different contexts. This study has made a start by examining the complex adaptive behaviour of young adults with diabetes, epilepsy and other long‐term conditions. However, further research is required to examine whether young adults with an even wider range of conditions, as well as adults and older people, share similar concepts of mastery and approaches to ruling their condition compared to those in this study.

Our findings also have several implications for healthcare policy and practice. One issue is whether there is sufficient emphasis in current policy and practice in England on helping young people and young adults to achieve their goals of autonomy as well as control. Over the last decade there has been a gradual shift in national and international policy, from a traditional focus on compliance and adherence, towards concordance (NPC Plus 2007, World Health Organization 2003). With the growing accent on concordance, patients and healthcare professionals are encouraged to reach a shared understanding of how best to manage their condition, taking into account the patient's personal preferences, beliefs and situation.

Despite these shifts in policy, the evidence from this study, based on data collected in 2006–2008, suggests that these new ideas have not been translated into practice. From the participants’ reports of their experiences, services were still focused more on promoting control than on autonomy. This is not ideal because, as we have shown, some young adults can achieve good control without feeling that they rule their condition or have much autonomy, having been overtaken by the demands of their regime or by the side‐effects of their medications. Others with an intractable form of their condition can, despite positively engaging with services, be left with little support to live as normal a life as possible with their uncontrolled condition. Those who selectively adhere to or otherwise resist their regime in an endeavour to retain their autonomy might also do so without discussing the risks with professionals. For these reasons, helping people to effectively manage their condition with the minimum of disruption and treatment burden (May *et al*. 2014) is important. Actively supporting people to achieve autonomy as well as to gain control might help them to feel more positive about living with their condition and avoid taking uninformed risks.

In addition, as we have shown, there are many factors that can influence young adults’ control and autonomy when they are growing up with a chronic illness. As Cooper and Geyer (2007, 2009) have argued, people with diabetes – and we would add other conditions too – are likely to benefit from approaches that help them to respond to change. By promoting their adaptive capacity, young people and young adults could be enabled to deal with the vagaries of their condition and life in general, especially in the ever‐changing landscape of adolescence. This contrasts with a more traditional healthcare approach where the focus is on achieving tight control as soon as possible and maintaining it by maximising adherence and minimising the risky behaviour that might jeopardise that control. That is not to say that the traditional approach does not work for some. However, it is less likely to help people to adapt in new and unpredictable situations, and to enable an acceptable balance in control and autonomy to be achieved and retained over time. Moreover, an unintended consequence of focusing on control might be to inspire rebellion in some young adults for whom the approach proves frustrating and unrealistic despite their best efforts.

Finally, the collection and dissemination of the young adults’ accounts forms an important online resource for informing and supporting their peers with similar conditions, as well as parents and healthcare professionals involved in their care. By participating in these studies and sharing their accounts of the mundane complexity of living with and managing a chronic illness, the young adults arguably helped to prepare and support others with the same condition, and their families and healthcare professionals, for the uncertainty they were likely to face. In many cases, they also demonstrated their capacity for positively adapting to it via the sorts of strategies they commended.
